# Discovery of Core Biotic Stress Responsive Genes in Arabidopsis by Weighted Gene Co-Expression Network Analysis

**DOI:** 10.1371/journal.pone.0118731

**Published:** 2015-03-02

**Authors:** Katherine C. H. Amrine, Barbara Blanco-Ulate, Dario Cantu

**Affiliations:** Department of Viticulture and Enology, University of California Davis, Davis, California, United States of America; Leibniz-Institute for Farm Animal Biology (FBN), GERMANY

## Abstract

Intricate signal networks and transcriptional regulators translate the recognition of pathogens into defense responses. In this study, we carried out a gene co-expression analysis of all currently publicly available microarray data, which were generated in experiments that studied the interaction of the model plant *Arabidopsis thaliana* with microbial pathogens. This work was conducted to identify (i) modules of functionally related co-expressed genes that are differentially expressed in response to multiple biotic stresses, and (ii) hub genes that may function as core regulators of disease responses. Using Weighted Gene Co-expression Network Analysis (WGCNA) we constructed an undirected network leveraging a rich curated expression dataset comprising 272 microarrays that involved microbial infections of Arabidopsis plants with a wide array of fungal and bacterial pathogens with biotrophic, hemibiotrophic, and necrotrophic lifestyles. WGCNA produced a network with scale-free and small-world properties composed of 205 distinct clusters of co-expressed genes. Modules of functionally related co-expressed genes that are differentially regulated in response to multiple pathogens were identified by integrating differential gene expression testing with functional enrichment analyses of gene ontology terms, known disease associated genes, transcriptional regulators, and *cis*-regulatory elements. The significance of functional enrichments was validated by comparisons with randomly generated networks. Network topology was then analyzed to identify intra- and inter-modular gene hubs. Based on high connectivity, and centrality in meta-modules that are clearly enriched in defense responses, we propose a list of 66 target genes for reverse genetic experiments to further dissect the Arabidopsis immune system. Our results show that statistical-based data trimming prior to network analysis allows the integration of expression datasets generated by different groups, under different experimental conditions and biological systems, into a functionally meaningful co-expression network.

## Introduction

Plants have evolved complex sensing, signaling, and defense mechanisms to cope with a broad range of pathogens [1]. Although some plant defense strategies are specific to the type of invading organism (e.g., fungi, bacteria) and its pathogenic lifestyle (i.e., biotrophic, necrotrophic, or hemibiotrophic), other responses are thought to be common to diverse biotic stresses [1,2]. Core responses to pathogens may result from cross-talk between hormone-related pathways [3,4], such as ethylene (ET), jasmonic acid (JA), salicylic acid (SA) and abscisic acid (ABA), and/or from signaling mediated by calcium [5,6], reactive oxygen species (ROS; [7]), and phosphorylation cascades [8]. Transcription factors convey these signaling cues and activate or repress the expression of genes involved in immune responses and metabolic processes.

Because most plant immune responses are under transcriptional control, transcriptome profiling approaches, including hybridization based microarray and RNA-seq, are effective tools to monitor at the genome-scale the activation or suppression of specific regulatory and metabolic pathways during the interactions between plants and microorganisms [9–11]. The expression profiles of individual genes can be integrated into co-expression networks where genes are clustered as a function of pairwise gene expression correlations [12]. Gene co-expression analyses have been conducted to reconstruct regulatory pathways [13], discover novel candidate genes [14,15], and identify key modulators of immune responses [16]. Genes belonging to the same co-expression sub-network (or module) are likely to be functionally related [17–19], participate in similar biological processes, or be part of the same pathway [13].

Gene co-expression networks have been shown to have small-world and scale-free properties [13,20,21], which are common topological properties of many other biological networks, including protein-protein interaction and metabolic networks [22]. In a scale-free network most nodes have few interactions, while few nodes, the hubs, are highly interconnected. In comparison to scale-free networks, random networks have a uniform number of edges per node [22]. A critical property of scale-free networks is that random perturbations do not alter the overall stability of the system, whereas perturbations to the most highly connected hubs are severely destabilizing [23]. The centrality to network architecture and high degree of connectivity of molecular hubs, including those in co-expression networks, tend to be associated with essential roles in biological processes [24–26]. Co-expression network hubs were also shown to evolve more slowly than genes with fewer co-expression partners, suggesting that changes in sequence or expression level of hub genes can be deleterious, which further supports their functional centrality [20].

In this study, we constructed an undirected weighted gene co-expression network leveraging a rich curated expression dataset comprising 32 publicly available microarray experiments that involved infections of Arabidopsis plants with a wide array of single-cell eukaryotic and bacterial pathogens with different parasitic lifestyles. We annotated the network components to identify modules enriched in genes known to be associated with disease responses. We applied a permutation analysis to determine whether the observed functional enrichments were significant. Network topology was then analyzed to identify intra- and inter-modular hub genes, which are potentially key components of the core responses that Arabidopsis activates when challenged by multiple and diverse biotic stresses.

## Results and Discussion

### Data collection and processing

**Fig 1:**
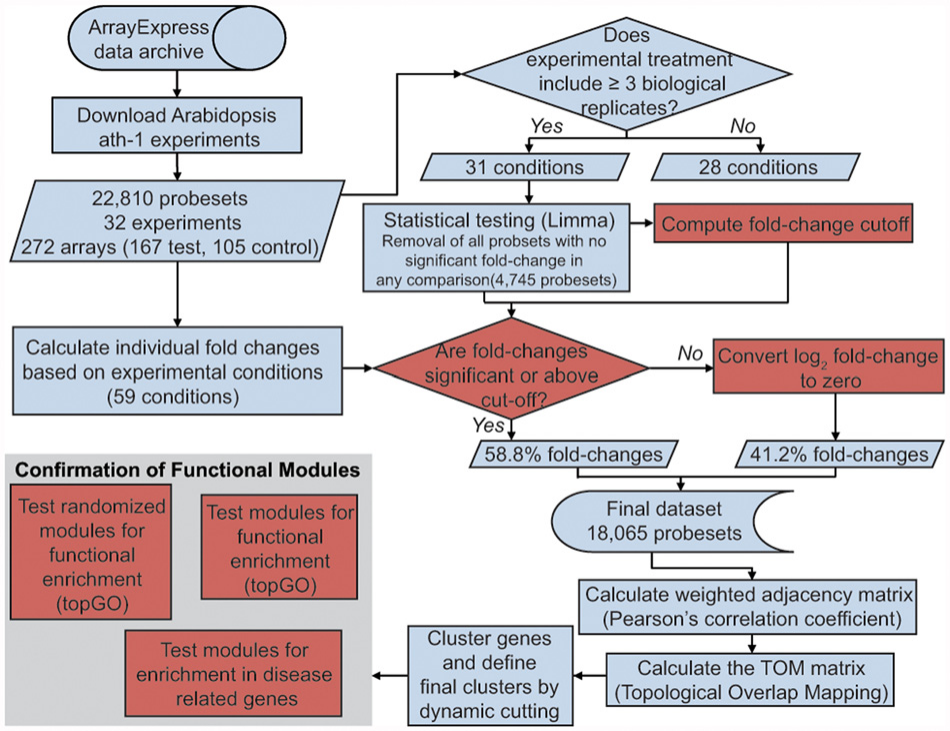
Flowchart of data collection, processing and analysis. A total of 272 arrays were used comprising 167 infected and 105 control arrays. Data were processed according to the number of biological replicates as shown. A total of 18,065 probesets was included in the final dataset with 58.8% of the values retained and 41.2% replaced with zero. Data were then partitioned into modules based on the Topological Overlap Matrix (TOM) values calculated with WGCNA [27]. Modules were tested for functional and disease-related enrichment using topGO [79]. Shapes in red depict methods unique to the work described in this paper.

Fig. 1 provides a schematic workflow of the analysis, from data collection and processing, network construction, to module identification and characterization. All publicly available microarray experiments involving wild-type Arabidopsis plants challenged with biotic agents were collected generating a comprehensive dataset that included multiple infectious agents (Table 1; S1 Table). Additional variation across experiments included different Arabidopsis accessions, infected tissues, and developmental stages (S1 Table). Hierarchical clustering of the 167 infected microarrays based on gene expression fold-changes revealed strong similarities between biological replicates, with no other experimental condition driving sample clustering across experiments (S1 Fig.).

**Table 1:** Summary of experiments included in this study.

Number of microarrays ^1^	Infection type^2^	Infectious agents^3^
**70**	BB	*Agrobacterium tumefaciens* (21), *Pseudomonas syringae* (49),
**7**	BH	*Xanthomonas campestris*
**4**	BN	*Ralstonia solanacearum*
**32**	FB	*Blumeria graminis* (8), *Glovinomyces cichoracearum* (16), *Fusarium oxysporum* (4), *Golovinomyces orontii* (4),
**21**	FN	*Alternaria brassicicola* (3), *Botrytis cinerea* (6), *Rhizoctonia solani* (6), *Sclerotinia sclerotiorum* (6)
**27**	OB	*Hyaloperonospora arabidopsidis* (14), *Hyaloperonospora parasitica* (5), *Phytophthora parasitica* (8),
**6**	PR	*Plasmodiophora brassicae*

^1^ Number of ATH1 arrays that were hybridized with cDNA from pathogen-infected samples

^2^ BB: bacterial biotrophs; BH: bacterial hemibiotrophs; BN: bacterial necrotrophs; FB: fungal biotrophs; FN: fungal necrotrophs; OB: oomycete biotrophs; PR: protist.

^3^ Values in parenthesis correspond to the number of arrays for each pathogen.

To identify genes responsive to pathogen infections, significant differential expression (DE) was tested for each probeset within each experiment as described in the Materials and Methods section. DE testing was applied to all experimental conditions that had at least three biological replicates of the healthy control and infected samples. The distribution of fold-changes corresponding to significant DE probesets was used to identify genes potentially differentially expressed in those experiments with less than three biological replicates, while the 4,745 probesets that did not display significant fold-changes in any of the experiments were excluded from the final dataset (Fig. 1). These excluded probesets were (i) housekeeping genes, (ii) genes that are not differentially expressed in response to any of the pathogens, and (iii) genes with large variation in expression between biological replicates, which may have masked differential regulation. To remove potential noise from the dataset, all comparisons that were not called significant or outside the fold-change thresholds were converted to a log_2_ fold-change value of zero. This approach ensured that weak fluctuations of expression were more likely to be true biological signal instead of measurement error or bias uncorrected by gcrma normalization.

### Construction of the co-expression network of pathogen responsive genes

A gene expression correlation network was constructed with the Weighted Gene Co-expression Network Analysis (WGCNA) method implemented in the WGCNA R package [27]. WGCNA identifies modules composed of genes that are connected based on the topological overlap mapping metric (TOM), a neighborhood proximity measurement that quantifies the degree of shared network neighbors [12]. Unlike unweighted network approaches where gene connections are dichotomized based on *a priori* selected correlation cutoff, weighted networks do not depend on a hard-threshold. Given the complexity of the multi-infection dataset, using a hard threshold would likely result in loss of information and sensitivity [28]. Therefore, a soft-thresholding power of 5 with a scale-free model fitting index *R* ^*2*^ > 0.688 (S2 Fig.) was applied to (i) maximize scale-free topology, while (ii) maintaining a high mean number of connections and (iii) eliminating small correlations.

**Fig 2:**
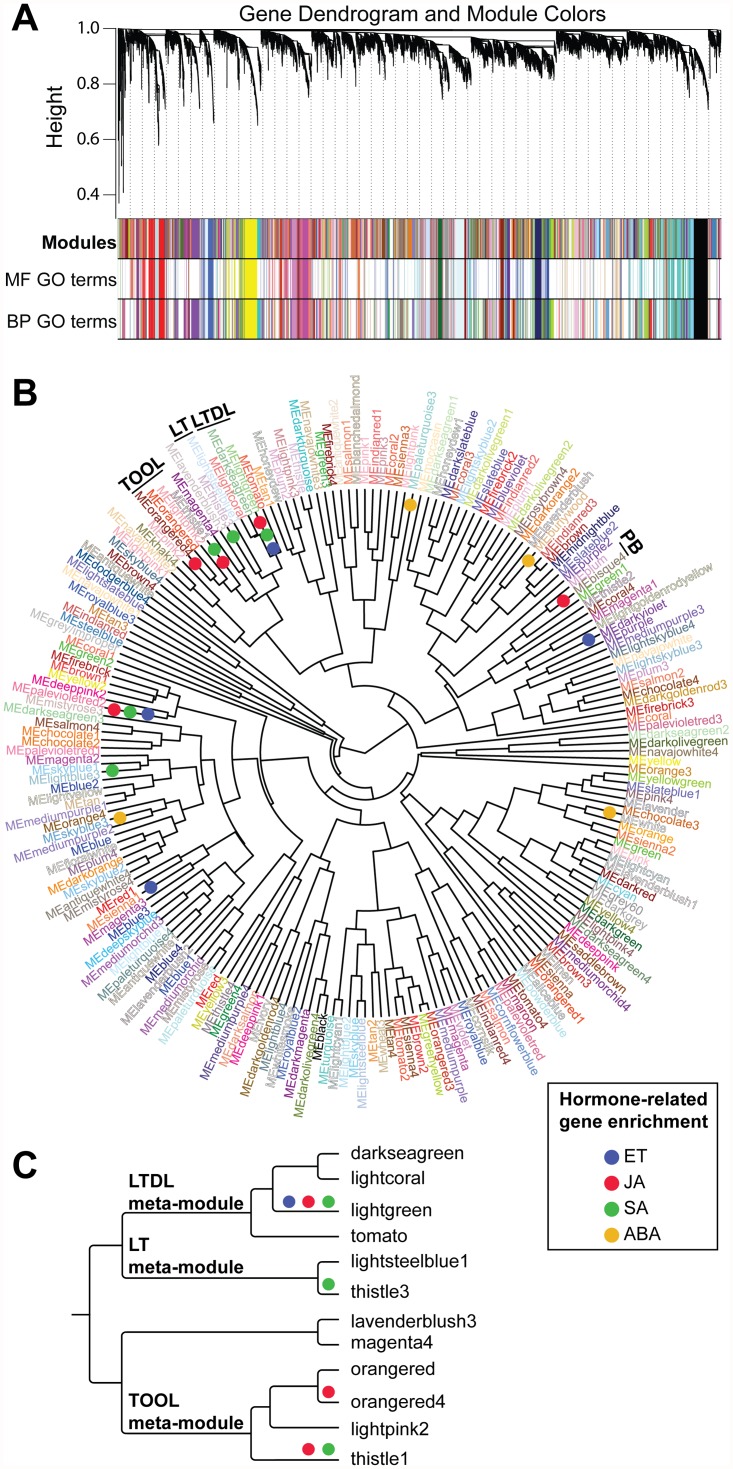
Graphical visualization of the Arabidopsis co-expression network. (A) Dendrogram of all differentially expressed probesets clustered based on a dissimilarity measure (1—TOM). Each line of the dendrogram corresponds to a probeset. The first multi-color bar below the dendrogram shows the 205 modules identified using the dynamic cutting method with each gene color-coded based on module assignment. The second and third multi-color bars highlight the modules enriched (*P*-value < 0.01) in molecular function (MF) and biological process (BP) GO terms. Each line corresponds to genes in modules enriched in GO terms, while line colors identify module membership. Module gene members are not always adjacent to each other because WGCNA modules do not comprise only leaves with their direct ancestors. (**B**) Circular tree showing hierarchical clustering of the 205 module eigengenes. Modules that are enriched in genes associated with hormones (ET: ethylene, JA: jasmonic acid, SA: salicylic acid, and ABA: abscisic acid) and that are part of higher-order (meta) modules discussed in the text are highlighted. (**C**) Hierarchical cluster tree showing the relationship between the TOOL, LTDL, and LT meta-modules based on correlations between their respective eigengenes. Hormone enrichment is also depicted in the tree.

As a result, an undirected weighted network with scale-free topology composed of 205 modules of Arabidopsis genes with correlated expression during pathogen infections was obtained (Fig. 2a; S2 Table). WGCNA assigned to each module a unique color label that was used as specific module identifier in the analyses described below [27]. The modules were composed on average of 87.7 genes (median gene number per module = 44), with the largest module (‘turquoise’) containing 802 genes. Four probesets were not grouped in any of the 205 modules and were added to the ‘grey’ (or improper) module.

To allow additional graph-theoretical calculations, including the identification of gene hubs (see below), the weighted network was converted into an unweighted network preserving all connections with TOM > 0.1 [29]. The unweighted network displayed a hierarchical and modular structure as suggested by the empirical distribution of the probability (*P*(*k*)) of finding nodes containing *k* edges (S3 Fig.). The properties of the unweighted co-expression network included a mean path length (distance between any two nodes) of 3.8 and mean clustering coefficient of 0.80, indicating that the network also had small-world characteristics [22]. The most highly connected nodes (top 5%) from both weighted and unweighted (TOM > 0.1) networks corresponded to members of multiple modules (46 and 38, respectively).

Computing correlations between modules indicated the presence of a higher-order structure in the unweighted co-expression network. Inter-modular correlations were determined by correlation analyses of the module eigengenes (i.e., the first right-singular vector of TOM values of a given module), which have been shown to be robust indicators of entire module trends [25]. One hundred fifty-two modules (75%) were merged into 30 meta-modules based on pairwise eigengene adjacency (*a*_*eigen I*,*J*_> 0.75); see Materials and Methods; Fig. 2b). The meta-module sizes ranged from 46 genes (2 modules) to 2,460 genes (16 modules; median meta-module size: 170.5 genes, 3 modules). Some meta-modules of interest include the TOOL (‘thistle1’, ‘orangered’, ‘orangered4’, ‘lightpink2’), LTDL (‘lightgreen’, ‘tomato’, ‘darkseagreen’, ‘lightcoral’), LT (‘lavenderblush3’, ‘thistle3’), and PB (‘plum’, ‘bisque4’) meta-modules, all enriched for disease response functions (Fig. 2b, c).

### Evaluation of module significance by gene ontology term enrichment

**Fig 3:**
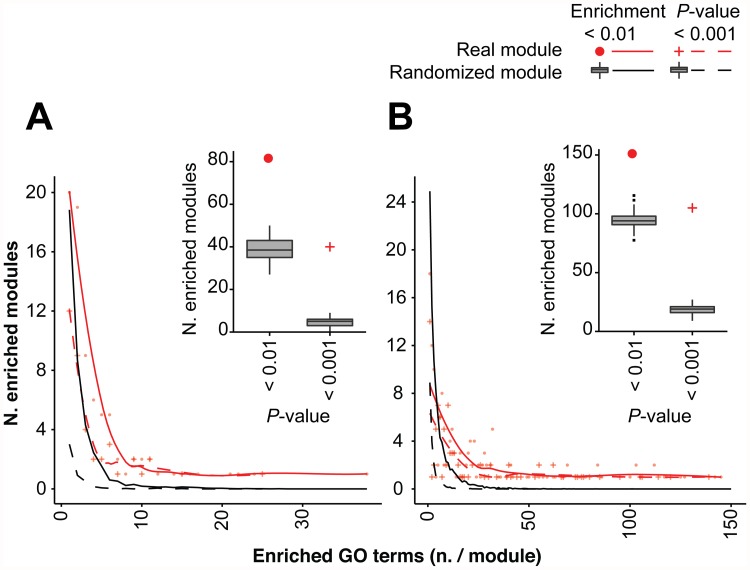
Gene Ontology (GO) term enrichments in modules identified by WGCNA and in randomly generated modules. (**A**) Molecular function and (**B**) biological process GO terms. Line plots represent the number of enriched modules as a function of the number of enriched terms in a module. Red symbols represent the observed counts, and red lines represent the best smooth of the data points. The black lines and symbols represent the mean number of enriched modules for modules with any given number of enriched GO terms in 100 random replicates. The boxplot represents the number of total modules with at least one enriched term in all 100 random permutations of the network. Red symbols represent the number of modules with at least one enriched term in the natural network. Real BP data included eight modules enriched for up to 274 terms, which were not shown in the figure.

Enrichment analyses of gene ontology (GO) terms within modules and meta-modules were conducted [30] to provide a biological interpretation of the constructed gene network. One hundred and fifty-one modules were enriched (*P-*value < 0.01) in GO terms related to specific Biological Processes (BP), while 82 modules showed over-representation (*P-*value < 0.01) of Molecular Function GO terms (MF; Fig. 2a and S3 Table). To validate the significance of the observed GO term enrichments, the same analysis was carried out in 100 randomly generated networks (Fig. 3). The permutation was conducted with random assignment of probesets to modules of the same size as in the WGCNA-created network. For both BP (Fig. 3a) and MF (Fig. 3b) GO terms, the number of enriched modules in the “real” network was significantly higher (BP: *P*-value *=* 0; MF: *P*-value = 0) than in the random networks. In addition, a significantly higher number of enriched GO terms (BP: *P-*value *=* 0; MF: *P*-value = 0) was found in the “real” network when compared to the random networks.

The modules with the largest number of enriched terms included enriched GO categories associated with biotic stress responses, signaling, and transcriptional regulation (S3 Table). The ‘orangered4’ module, composed of 122 genes and a member of the TOOL meta-module, had an over-representation of BP terms related to immune and defense responses (e.g., GO:0002376, GO:0045087. GO:0006955, GO:0006952, GO:0031347, and GO:0030968). This module also displayed a high number of enriched MF categories (38 terms) and most of them were related to signaling and stress responses, such as “protein kinase activity” (GO:0004672; GO:0004674), “phosphotransferase activity” (GO:0016773) and “nucleotide binding” (GO:0032559; GO:0030554; GO:0017076). Significant differential expression was detected in 60% of the 20,374 comparisons between control and infected samples (122 probesets x 167 experiments) that compose the ‘orangered4’ module, with (77.6%) of the fold changes significantly up-regulated by both biotrophs and necrotrophs (S4 Fig.). These results suggest that members of the ‘orangered4’ module are part of a general biotic stress response that Arabidopsis activates when challenged by different types of pathogens.

The ‘thistle1’ module, another member of the TOOL meta-module, also included genes associated with plant responses to biotic stress, such as “responses to chitin” (GO:0010200), a pathogen-associated molecular pattern. This module was also enriched in MF terms related to transcription factor activities (GO:0003700 and GO:0001071). Nineteen percent of the members of ‘thistle1’ were significantly up-regulated in all comparisons between healthy and pathogen infected samples; these included genes expected to be involved in signaling and responses to pathogen infections, such as WRKY and WRKY-associated transcription factors (At4g23810, probeset 254231_at, and At1g80840, probeset 261892_at), calcium binding-proteins [5] (At5g54490, probeset 248164_at; At2g41100, probeset 267083_at), a FAD-binding Berberine family protein (At4g20830, probeset 254432_at), and JA biosynthesis and signaling (At1g72520, probeset 260399_at; At1g19180, probeset 256017_at). Members of ‘thistle1’ and ‘orangered4’ were also found co-expressed in another Arabidopsis disease response co-expression study using different methods, further supporting the TOOL meta-module creation, and its enrichment for disease response activity [16].

Not all modules were enriched in GO terms directly associated with responses to disease, suggesting that transcriptional reprogramming in response to pathogens involves other metabolic processes besides defense reactions. For example, the ‘darkgreen’ module displayed the largest number of enriched BP terms (215 terms at *P* < 0.001), with the most significant ones related to photosynthesis and growth-related processes (GO:0015979, GO:0019684 and GO:006091). The only MF term that was significantly enriched in the ‘darkgreen’ module was the tetrapyrrole binding activity (GO: 0046906), further suggesting that this module is involved in photosynthesis. About half of the comparisons (45.4% of 32,899) in the ‘darkgreen’ module show DE in response to biotic stresses and most of them (74%) down-regulated (S4 Fig.). Among the ‘darkgreen’ genes, At5g64040 (probeset 247320_at) that encodes a subunit of photosystem I reaction center displayed the greatest down-regulation across all infections analyzed (median log_2_ fold change: -0.86; S2 Table). Global down-regulation of photosynthetic genes is considered a common plant response to pathogen and insect attack [31–35]. Inhibition of the photosynthetic activity causes a switch from primary metabolism towards non-assimilatory (e.g., carbon-consuming) pathways, which can enhance the production of anti-microbial metabolites and the expression of defense-related genes [36].

### Identification of modules enriched for known pathogen responsive genes

To further explore the functional enrichment in the 205 co-expression modules, genes involved in plant hormone networks were identified based on the annotations of the Arabidopsis Hormone Database (AHD). The AHD includes 1,318 genes encoding proteins involved in the biosynthesis, modification, and signaling of plant hormones; most of which are experimentally supported by forward and/or reverse genetic evidence [37]. An emphasis on ET, SA, JA and ABA pathways was given in this study, as they play crucial roles during plant-microbe interactions [38,39]. Among the 18,065 probesets that compose the network, 133, 104, 132 and 314 probesets were associated with ET, SA, JA, and ABA pathways, respectively [40]. A total of 179 DE probesets were assigned to multiple signaling pathways by AHD annotations [37]. Hormone enrichment was tested with the classic Fisher’s exact test. Modules significantly enriched (*P*-value < 0.01) in ET (4 modules), SA (5 modules), JA (5 modules), and ABA (4 modules) associated genes were identified (S4 Table). The ‘lightgreen’ and ‘palevioletred2’ modules were enriched in genes from three hormone pathways including ET, SA and JA (Fig. 2b). Importantly, all the modules enriched in ET, SA and/or JA hormone-related genes were also enriched in other genes with GO terms associated with responses to pathogens further supporting the validity of the constructed network. Five of these enriched modules were part of four meta-modules of co-expressed genes (i.e., TOOL, LTDL, LT, and PB; Fig. 2b, c). For example, ‘orangered4’ and ‘thistle1’ had an over-representation of hormone-associated genes (Fig. 2c; S4 Table) and in GO terms related to signaling and disease responses (see previous section). Not all the members of the modules enriched in hormone-related genes have AHD annotations; however some genes could potentially be part of the hormone networks. Functional coherence between co-expressed genes can be exploited to assign putative functions to the genes inside these modules that are still reported as coding proteins with “unknown function”, such as those provided in S5 Table.

Besides hormone-related functions, module enrichment in other well-characterized responses to pathogen infection were also evaluated, such as those involving genes coding for pathogenesis-related (PR) proteins [41]. PR proteins have diverse functions in plant defense, including signaling (e.g., PR-1), hydrolysis of fungal cell walls (e.g., PR-2 and PR-4) and contact toxicity (e.g., PR-5 [42]). Remarkably, *PR-1* (At2g14610, probeset 266385_at), *PR-2* (At3g57260, probeset 251625_at), and *PR-5* (At1g75040, probeset 259925_at) were tightly co-expressed and belonged to the ‘coral4’ module, which was enriched in 67 BP terms, including “immune system process” (GO:0002376). ‘Coral4’ formed a clade in the eigengene dendrogram (Fig. 2b) with the PB meta-module and the ‘thistle2’ and ‘green1’ modules. *PR-4* (At3g04720, probeset 258791_at) was part of the ‘darkviolet’ module, which was enriched in ET pathway-associated genes (Fig. 2b, S3 Table). A more extensive list of experimentally verified disease response-related genes, and the modules they are associated with, are available in S6 Table.

Modules were also analyzed for enrichment in specific transcription factor (TF) families. TF families that control the expression of defense-related genes, hormone-dependent or independent, include, among others, the ethylene-responsive-element-binding factor (ERF), basic-domain leucine-zipper (bZIP), WRKY, and MYB proteins [43]. The ‘black’ module was enriched (*P*-value < 0.01) for TF families, particularly APETALA2 (AP2)/Ethylene-Responsive Element Binding Proteins (EREBP), Homeobox, MADS, MYB, NAC, and bZIP (S4 Table). Most of these genes displayed small fold-changes in expression as a consequence of pathogen infection (S2 Table). Of the 25.7% differentially regulated points in the ‘black’ module, 53% were down-regulated in response to pathogens (S4 Fig.). Given that transcription factors generally have complex relationships including interactions with themselves, the genes they regulate, and other transcription factors (e.g., auto-regulation, feed-forward loops, multi-component loops, regulator chains, single input chains, and multiple input chains; [44,45]), it is likely that direct correlations in co-expression networks cannot be used to identify transcription factor relationships [46].

A similar approach was used to determine if the identified modules were significantly enriched in particular *cis*-regulatory elements using annotations from the Arabidopsis *cis*-regulatory element database (AtcisDB; [47,48]). The meta-module LTDL, characterized by a significant overrepresentation of genes associated with the ET and SA pathways (Fig. 2b, c), was also enriched in motifs known to be under hormone regulation. These included the ET/JA-responsive motifs, G-box (99 genes; *P =* 0.012) and GCC-box (55 genes; *P =* 0.02; [49,50]). In addition, W-box motifs (264 genes; *P =* 0.02), which are regulated by SA-induced WRKY TFs, were present in this meta-module [51–53]. The W-box motif is present in the promoter of a large variety of genes involved in immune responses [54–56], even several WRKY factors themselves contain this *cis*-regulatory element [57]. An enrichment in W-box motifs was also found in the LT meta-module (98 genes, *P* = 0.02), which contains ‘thistle3’, a module enriched for SA-associated genes (see previous section). The ABA-responsive elements, ATHB1 (9 genes, *P* = 0.03), and DPBF1&2 binding site motifs (94 genes, *P* = 0.01), were significantly enriched in the LT meta-module as well. Both motifs were shown to be present in genes with functions in stress tolerance [58].

### Network topology of the four disease responsive and hormone-associated gene enriched meta-modules

**Fig 4:**
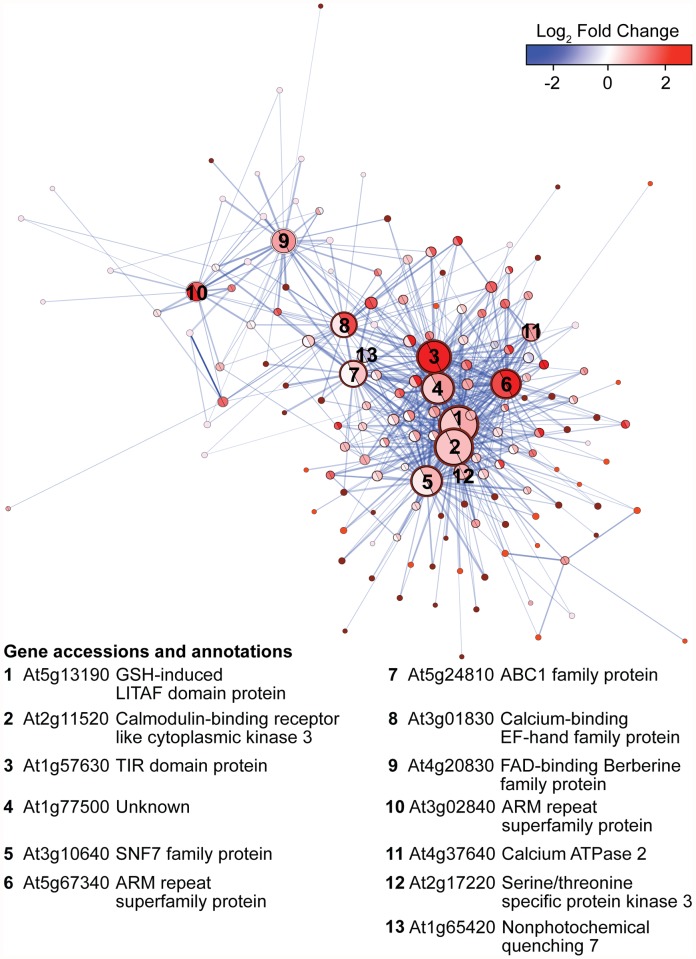
Graphical representation of the TOOL meta-module. Nodes of the unweighted network (TOM > 0.1) are visualized as circles and correlations between nodes as lines (edges). Node size is proportional to degree of unweighted connectivity; the colors of the edge of the nodes correspond to module membership; edge width and opacity are proportional to TOM and adjacency values between the two connected nodes, respectively. The central color of each node is based on the mean expression log_2_ fold-change (up-regulation in red, down-regulation in blue) in response to necrotrophs (left) and biotrophs (right). The shape of the network is based on a force-directed graph calculation which creates a shape based on centrality computed with Cytoscape v. 2.8 [29]. Intra-modular hub genes are numbered according to their ranked unweighted connectivity.

To determine the local co-expression network topology, and to identify the main hub genes within the four hormone-enriched and disease-related meta-modules, the connectivity of each meta-module was evaluated (Table 2). The TOOL meta-module displayed a non-random network topology (Table 2) with most (92%) of the top 5% of connected genes up-regulated by both biotrophic and necrotrophic infections (Fig. 4, S7 Table). Fifty-six percent of the probesets in the TOOL meta-module were strongly connected (TOM > 0.1). These connected nodes consisted of 94 ‘orangered4’, 37 ‘thistle1’, 22 ‘orangered’, and 1 ‘lightpink2’ probesets. The three most connected genes in the TOOL meta-module corresponded to (i) At5g13190 (probeset 250289_at, ‘orangered4’ module), which encodes a LPS-Induced Tumor necrosis Alpha Factor (LITAF) domain protein shown to negatively regulate programmed cell death [59], (ii) At2g11520 (probeset 263274_at, ‘orangered4’ module) encoding a cytoplasmic calmodulin-binding receptor-like kinase [60], and (iii) At1g57630 (probeset 246405_at, ‘orangered4’ module) encoding a transmembrane Toll-Interleukin Receptor (TIR) domain protein [61]. The transmembrane pathogen receptor At1g57630 was not only one of the most highly connected nodes in the TOOL meta-module, but was also up-regulated by most of the experiments (75%) and displayed the highest median fold-change across experiments (log_2_ fold-change: 2.44).

**Table 2:** Summary of network properties of the TOOL, LTDL, LT, and PB meta-modules.

Module	TOOL	LTDL	LT	PB
**Module size** ^**1**^	277 (155)	345 (145)	167 (111)	156 (59)
**Density**	0.07	0.04	0.07	0.06
**Centralization**	0.08	0.08	0.09	0.08
**Heterogeneity**	0.50	0.80	0.42	0.60
**Mean clustering coefficient**	0.12	0.10	0.10	0.11
**Mean connectivity**	19.50	13.82	10.95	8.73
**Factorizability**	0.70	0.76	0.67	0.70

^1^ Numbers in parenthesis correspond to the number of nodes with at least one connection with TOM > 0.1.

The LTDL meta-module also displayed non-random network topology (Table 2) with the top 5% of connected (81.2%) genes up-regulated in response to biotrophic and necrotrophic pathogens (S5 Fig. a). Most interconnected nodes were members of the ‘lightgreen’ (92), ‘lightcoral’ (35), ‘darkseagreen’ (15), and ‘tomato’ (2) modules. The three most connected nodes were (i) At1g61370 (probeset 264756_at, ‘lightgreen’ module) encoding a S-locus lectin protein kinase that, (ii) At3g55470 (probeset 251790_at, ‘lightgreen’ module) coding for a calcium-dependent lipid-binding (CaLB domain) family protein, and (iii) At3g25250 (probeset 257840_at, ‘lightgreen’ module) encoding the oxidative signal-inducible kinase 1 (OXI1); all of these genes have been associated with defense signaling [62–66]. In particular, OXI1 is essential in the signal transduction pathway linking ROS signaling and downstream responses to biotic and abiotic cues [66].

In the LT meta-module (Table 2; S5 Fig. b), the three most connected nodes were At5g58730 (probeset 247788_at; ‘lightsteelblue1’ module) coding for a myo-inositol kinase and two genes encoding proteins involved in defense responses by callose deposition, At4g12120 (probeset 254857_at, ‘lightsteelblue1’ module) and At1g15430 (probeset 262571_at, ‘lightsteelblue1’ module)]. Most top hub nodes in the PB meta-module (Table 2; S5 Fig. c) did not have functions obviously linked to pathogen responses, but included a gene coding for the UDP-dependent glycosyltransferase UGT76B1 (At3g11340, probeset 256252_at, ‘plum’ module), which was shown to be involved in resistance against *P*. *syringae*, a biotrophic bacterium, and susceptibility to *A*. *brassicicola*, a necrotrophic fungus [67].

### Identification of hub genes with putative roles in responses to biotic stress

**Fig 5:**
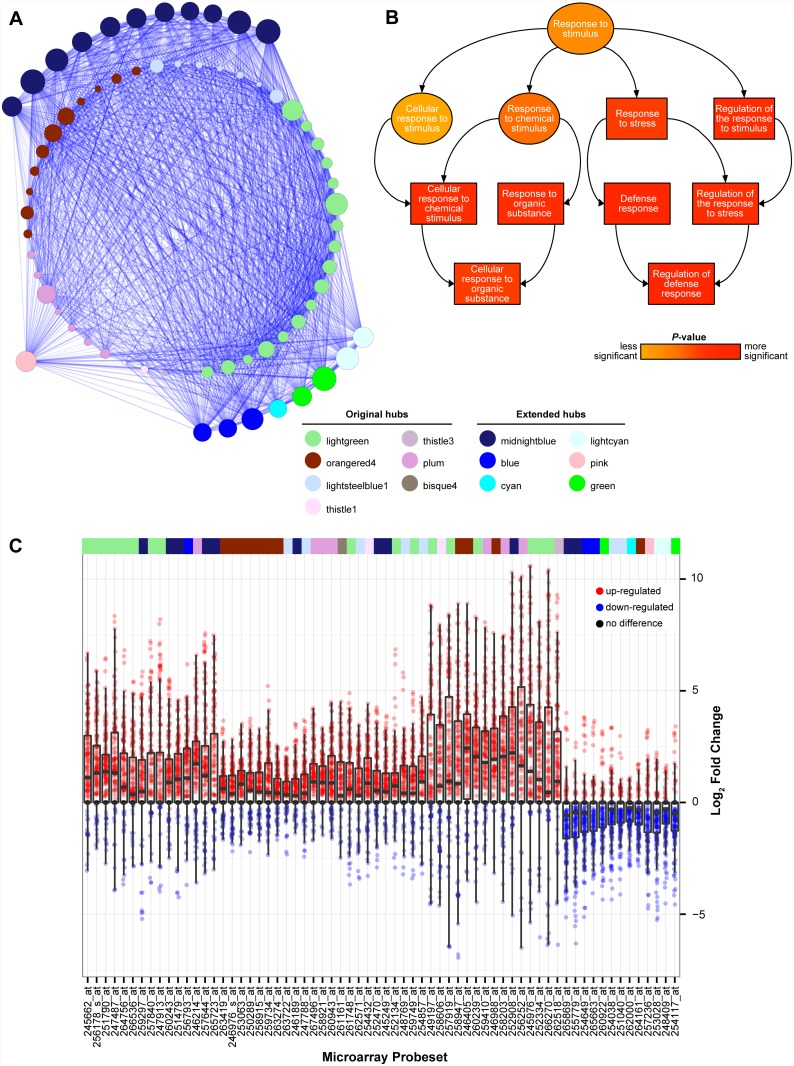
Characterization of the disease response hub genes extracted from the Arabidopsis co-expression network. (**A**) Unweighted network showing the connectivity (TOM > 0.1) between the 46 hub genes identified in the LTDL, PB, TOOL, and LT meta-modules and 20 extended hub genes (S7 Table). Nodes are visualized as circles and correlations between nodes as lines (edges). Node size is proportional to degree of unweighted connectivity; the colors of the nodes correspond to modular assignments. The inner circle represents the disease response-related meta-module hub connectivity. The outer circle represents the extended hub genes found to have TOM > 0.1 connections with > 75% of the original hubs. (**B**) Gene Ontology-annotated hierarchy of the top 5% enriched biological processes in the 66 hub genes. Boxes represent direct assignments of GO terms to hub genes, and ellipses represent parent terms assigned by topGO during analysis. (**C**) Boxplot showing the distribution of gene expression fold changes (log_2_) of the hub nodes in each microarray. The modules associated to each of the genes are provided in the multi-color bar. Red and blue dots represent comparisons in which genes were up- and down-regulated, respectively. For details refer to S7 Table.

Unweighted node connectivity information was used to identify hub genes within the four defense-related meta-modules, LTDL, PB, TOOL, and LT (S4 and S7 Tables). Nodes were ranked by their connectivity and 46 putative hub genes corresponding to the top 5% of the most connected nodes within each meta-module were selected for further analysis (S7 Table). Calculations of betweenness centrality (a measure of how important a node is for joining other connections in the network) confirmed that these hub genes had the highest overall intra-modular centrality (S7 Table) [68]. Of these 46 hub genes, 37%, 17.4%, 28.2%, 17.4% were members of the LTDL, PB, TOOL, and LT meta-modules, respectively. These hub genes were highly inter-connected (TOM > 0.1) to at least one other hub gene from the same or a different meta-module (Fig. 5a). High interconnectivity between the hub genes implies that the processes they are involved in are potentially co-regulated. The most interconnected hub gene with 41 strong connections (TOM > 0.1) was At1g71100 (probeset 259749_at; ‘lightgreen’ module), a gene encoding a putative ribose 5-phosphate isomerase (RSW10 [69]).

Further exploiting the property of high inter-connectivity between the hub genes, the co-expression network was mined for genes that were connected to more than 75% (TOM > 0.1) of the hub genes from the four meta-modules analyzed. Twenty additional hub genes from six other modules were identified; the majority belonged to the ‘midnightblue’ module (55%), followed by ‘blue’ (15%), ‘green’ (10%), ‘lightcyan’ (10%), ‘cyan’ (5%) and ‘pink’ (5%) modules (Fig. 5a).

In the final set of 66 hub genes, we found a significant enrichment in responsive functions to biotic stress (Fig. 5b, S8 Table). The top enriched BP terms were associated with immune system processes and defense responses (GO:0002376 and GO:0006952), and their regulation (GO:0002682). Remarkably, most hub genes (80.3%) were up-regulated by all types of pathogens (Fig. 5c, S7 Table), which suggests that they may represent core components of Arabidopsis responses to biotic stress. The proteins encoded by the top 15 highest expressed genes (among all types of pathogen infection; S7 Table) included: (i) the WRKY15 TF (AT5G13080, probeset 245976_at, ‘lightgreen’ module), which was shown to be involved in Arabidopsis resistance to necrotrophic and biotrophic pathogens [70,71], (ii) *UGT76B1* (mentioned in the previous section), a known modulator of SA/JA-dependent pathways [67], (iii) the calmodulin-like protein CML37 (At5g42380, probeset 249197_at, ‘lightgreen’ module’), which is induced in response to abiotic and biotic stresses [72], and (iv) the MYB15 TF (At3g23250, probeset 257919_at, ‘lightgreen’ module) that is involved in ABA-mediated stress responses [73]. A smaller fraction of hub genes was down-regulated (19.7%) by both biotrophs and necrotrophs (Fig. 5c, S7 Table). Most of the down-regulated genes (75%) corresponded to genes involved in larger system-wide processes including photosynthesis and cell cycle control, such as the genes coding for the high chlorophyll fluorescence protein HCF243 (At3G15095, probeset 257236_at, ‘pink’ module) and the ribonucleotide reductase RNR1 (At5G02250, probeset 251040_at, ‘lightsteelblue1’ module) (S7 Table).

### Conclusions

In this study we applied a novel approach to describe the complexity of plant responses to biotic stresses at the transcriptional level by taking advantage of the large amount of expression data available for the model plant Arabidopsis. We show that statistical-based data trimming prior to weighted network analysis can be used to integrate expression datasets generated using different experimental approaches. This allowed us to identify modules of functionally related co-expressed genes whose transcriptional regulations are responsive to multiple pathogen infections. We show that functional enrichments in the identified modules are significantly greater than in randomly generated networks. We identified hub genes that can serve as candidates for reverse genetic studies because of their high degree of connectivity within and across meta-modules enriched for immune response functions. Co-expression associations reported here can be further exploited to infer potential functions for those genes coding for proteins with unknown activities.

## Materials and Methods

### Metadata collection and curation

Microarray data were obtained from ArrayExpress and imported into R (www.r-project.org/) in September 2013 using the ArrayExpress data loading package v. 1.20.0 [74]. All datasets used in this study were generated using the Affymetrix Arabidopsis ATH1 Genome Array (ATH1) platform (GEO accession number GPL198), which contains more than 22,500 probesets representing approximately 24,000 genes (http://www.ncbi.nlm.nih.gov/geo/query/acc.cgi?acc=GPL198). The final dataset included 272 microarrays comprising 52 comparisons (31 with more than 3 biological replicates) between pathogen-infected (167 microarrays) and control samples (105 microarrays). Bacterial (4 species), fungal (8 species), oomycete (3 species), and protist (1 species) pathogens were grouped based on their parasitic life style as biotrophs (10 species), hemibiotrophs (1 species) or necrotrophs (5 species; Table 1).

Prior to statistical testing, hybridization intensities were normalized across all experiments using gcrma v. 2.32.0 [75]. Differential expression for experiments that included at least three healthy control samples was tested with the Limma R package v. 3.16.8 [76]. All analyses described in this work were conducted on the 18,065 probesets, out of the total 22,810 spotted on the ATH1 array, whose expression was significantly changed at least by one pathogen infection treatment. The empirical probability distribution of the fold-changes associated with significant DEs (*P*<0.05) was used to define a log_2_ fold-change threshold (*α*_+_ = 0.2777194, *α*_ = -0.2784232) to identify probesets potentially differentially expressed in those experiments with less than three control biological replicates. To remove potential noise, all fold-change values associated with comparisons that were not considered significant by Limma or potentially significant by threshold-filtering were converted to ‘zero’, which in log_2_ scale corresponds to complete absence of differential regulation between treatments, with the function *f(C) = I*_*C*_ *(X)* where:

$$I_{C_{j}}\left(x_{i}\right)=\left\{ \begin{array}{l} 0\;if\;\alpha_{-}\;<\;x_{i}\;<\;\alpha_{+}\\ x_{i}\;if\;x_{i}\;<\;\alpha_{{}_{-}}\\ x_{i}\;if\;x_{i}\;>\;\alpha_{+} \end{array}\right.$$

In order to maintain one data point per biological replicate of the pathogen-treated samples, the retained fold-change values were calculated by subtracting the median normalized log_2_ expression values of healthy control samples from the respective pathogen-treated samples.

### Weighted co-expression network construction

Co-expression analysis was conducted using the Weighted Correlation Network Analysis (WGCNA) v. 1.3.6 [27,77]. Instead of creating multiple networks and comparing their modules, which would have required a significant reduction of the dataset due to the high heterogeneity of the final dataset (varying numbers of replicates, diverse infecting pathogens, different time points of infection…), we generated with basic WGCNA network creation functions (flashClust() and cutreeDynamic(), [27,77]) a single co-expression network that incorporated all filtered expression values. Multiple branch cutting approaches were tested and dynamic cutting with the ‘hybrid’ parameter was selected because hard threshold cutting has been shown to be too rigid for dendrograms with potentially complicated transcriptomic data [78]. The ‘dynamic’ cutting can identify nested clusters and has been shown to better detect outliers [78]. A threshold of minimum module size of 20 genes was imposed. For network visualization and topological analyses, the unweighted network was extracted using a hard threshold of TOM > 0.1 and imported into Cytoscape v. 2.8 [29].

### Module enrichment tests

Gene Ontology (GO) enrichment in modules was carried out with topGO v. 2.12.0 [79] using the annotation of the ATH1 array from the ath1121501.db R package v. 2.9.0 [41]. The classic Fisher’s exact test method was applied. Each module was tested for enrichment in terms of the Molecular Function (MF) and the Biological Process (BP) categories. GO terms with expected values of less than one gene (i.e., determined by chi-square test) were not included in the analyses, in order to eliminate false positives from ill-annotated terms.

Hormone-related gene and transcription factor enrichments were also determined with a classic Fisher’s exact test. The list of genes annotated as involved in hormone biosynthesis, signaling, or modification were obtained from the Arabidopsis Hormone Database (ATHD; [37]). Transcription factor annotations were obtained from the Plant Transcription Factor Database (PlnTFDB) [80]. All modules were tested individually for enrichment in each category (S4 Table).

The Fisher’s exact test was used to assess significant enrichment of each *cis*-regulatory element type independently with contingency tables consisting of total counts of element presence in the Arabidopsis genome (AtcisDB; [47,48]), total counts of element presence in meta-modules, total predicted *cis-*regulatory elements in the Arabidopsis genome, and total count of *cis-*regulatory elements in meta-modules.

### Module analysis

Module eigengenes were calculated for each module (defined as the first right singular vector of the module, constructed with the TOM matrix), calculated using the moduleEigengenes() function in WGCNA. Pairwise eigengene correlation distances defined as $a_{eigen\;I,J}=\frac{1+cor(E_{I},E_{J})}{2}$, where *I* and *J* are modules and *E*_*I*_ and *E*_*J*_ are vectors of eigengene values from each module, were used to cluster the modules to create a tree of putative relatedness of function based on module trend [12]. Modules were clustered in meta-modules based on the 0.25 cutoff (corresponding to a correlation of 0.75) for the module eigengene dendrogram (Fig. 2b). Cytoscape v. 2.8 [29] was used for network visualizations and to calculate topological properties. Cytoscape statistics are reported in S7 Table. Topologies were visualized using the ‘shortest path’ method after removing edges associated with TOM values < 0.1. Equation (1)

## Supporting Information

S1 Fig Hierarchical clustering tree of ATH1 microarray samples based on the expression fold-changes of differentially expressed probesets. Dendrogram tips are labeled with the ArrayExpress unique name and experiment identifier, followed by two-letters that identify the infection type (BB, bacterial biotroph; BN, bacterial necrotroph; BH, bacterial hemibiotroph; FB, fungal biotroph; FN, fungal necrotroph; OB, oomycete biotroph; PR, protist biotrophs). Same colors in the six bands below the dendogram depict microarray experiments that are part of the same experiments, or that involved the same infective species, strain, type of pathogen, Arabidopsis accession, or infected tissue type.
(PDF)

S2 Fig Scale free topology model fitting by trimmed data. **(A)** Plot showing the scale free topology *R* ^*2*^ values in function of increasing soft thresholding power. **(B)** Plot showing the relation between mean connectivity and soft threshold.
(PDF)

S3 Fig Empirical cumulative distribution function of unweighted gene connectivity. Empirical distribution of the probability (*P*(*k*)) of finding nodes containing *k* edges (nodes with connectivity of TOM > 0.1) indicates a hierarchical and modular structure.
(PDF)

S4 Fig Proportion of genes up-regulated in response to biotrophic and necrotrophic pathogens in each module of co-expressed genes. Black lines highlight the ten modules enriched in genes associated with hormone activity.
(TIF)

S5 Fig Force-directed network visualization of the LTDL (A), LT (B) and PB (C) meta-modules. Only nodes with at least one connection with TOM > 0.1 are visualized. Node size is proportional to degree of unweighted connectivity; the colors of the edge of the nodes correspond to module membership; edge width and opacity are proportional to TOM and adjacency values between the two connected nodes, respectively. The central color of each node is based on the mean expression fold-change (up-regulation in red, down-regulation in green) in response to biotrophs (left) and necrotrophs (right). The numbers of nodes correspond to the intra meta-module connectivity ranking (S7 Table).
(TIF)

S1 Table Microarray datasets used in this study. (XLSX)

S2 Table Module membership, connectivity, and expression pattern of all 18,065 probesets included in the study. (XLSX)

S3 Table Enriched GO terms in the co-expressed modules. (XLSX)

S4 Table Modules enriched in hormone-associated genes and transcription factors. (XLSX)

S5 Table List of genes encoding proteins with unknown function associated with phytohormonal meta-modules based on TAIR annotations as of August 2014. (XLSX)

S6 Table Module memberships of genes with known function in plant-microbe interactions. (XLSX)

S7 Table Description of the 66 hub genes. (XLSX)

S8 Table Detailed topGO-based Gene Ontology enrichment information for hub genes and meta-modules (LTDL, LT, TOOL, and PB). (XLSX)
