# Supplementary figures and images for: Discovery of Core Biotic Stress Responsive Genes in Arabidopsis by Weighted Gene Co-Expression Network Analysis
Source: PLoS One. 2015 Mar 2;10(3):e0118731. doi: 10.1371/journal.pone.0118731 (PMC4346582; doi:10.1371/journal.pone.0118731)

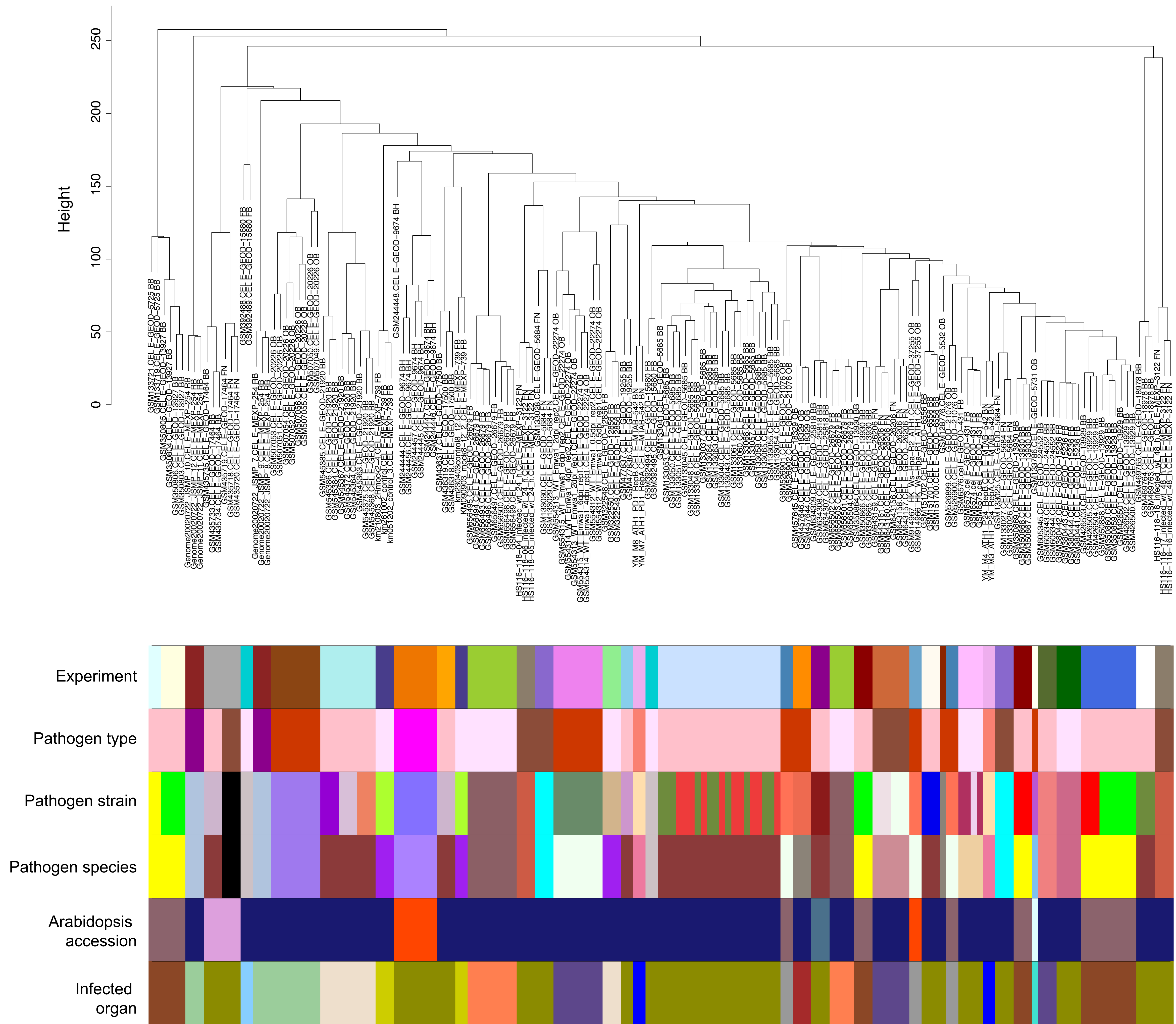

Supplement: S1 Fig — Dendrogram tips are labeled with the ArrayExpress unique name and experiment identifier, followed by two-letters that identify the infection type (BB, bacterial biotroph; BN, bacterial necrotroph; BH, bacterial hemibiotroph; FB, fungal biotroph; FN, fungal necrotroph; OB, oomycete biotroph; PR, protist biotrophs). Same colors in the six bands below the dendogram depict microarray experiments that are part of the same experiments, or that involved the same infective species, strain, type of pathogen, Arabidopsis accession, or infected tissue type. (PDF) [file pone.0118731.s001.pdf]

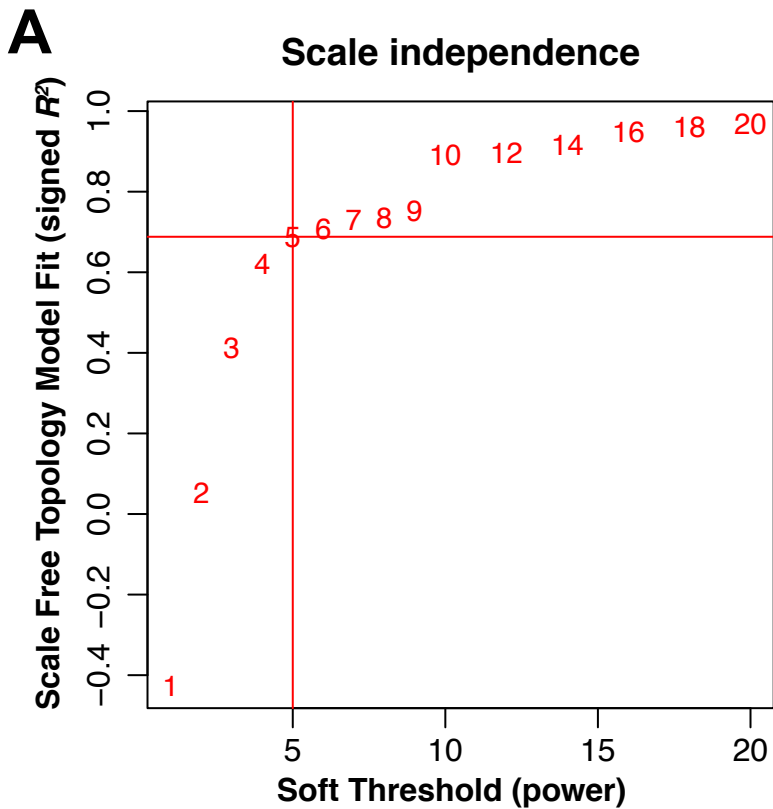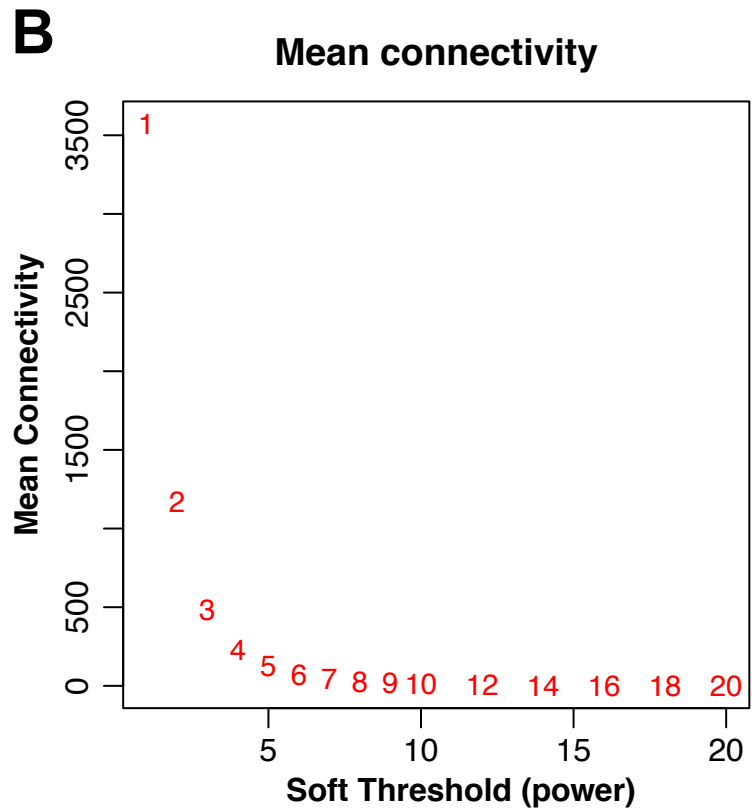

Supplement: S2 Fig — (A) Plot showing the scale free topology R 2 values in function of increasing soft thresholding power. (B) Plot showing the relation between mean connectivity and soft threshold. (PDF) [file pone.0118731.s002.pdf]

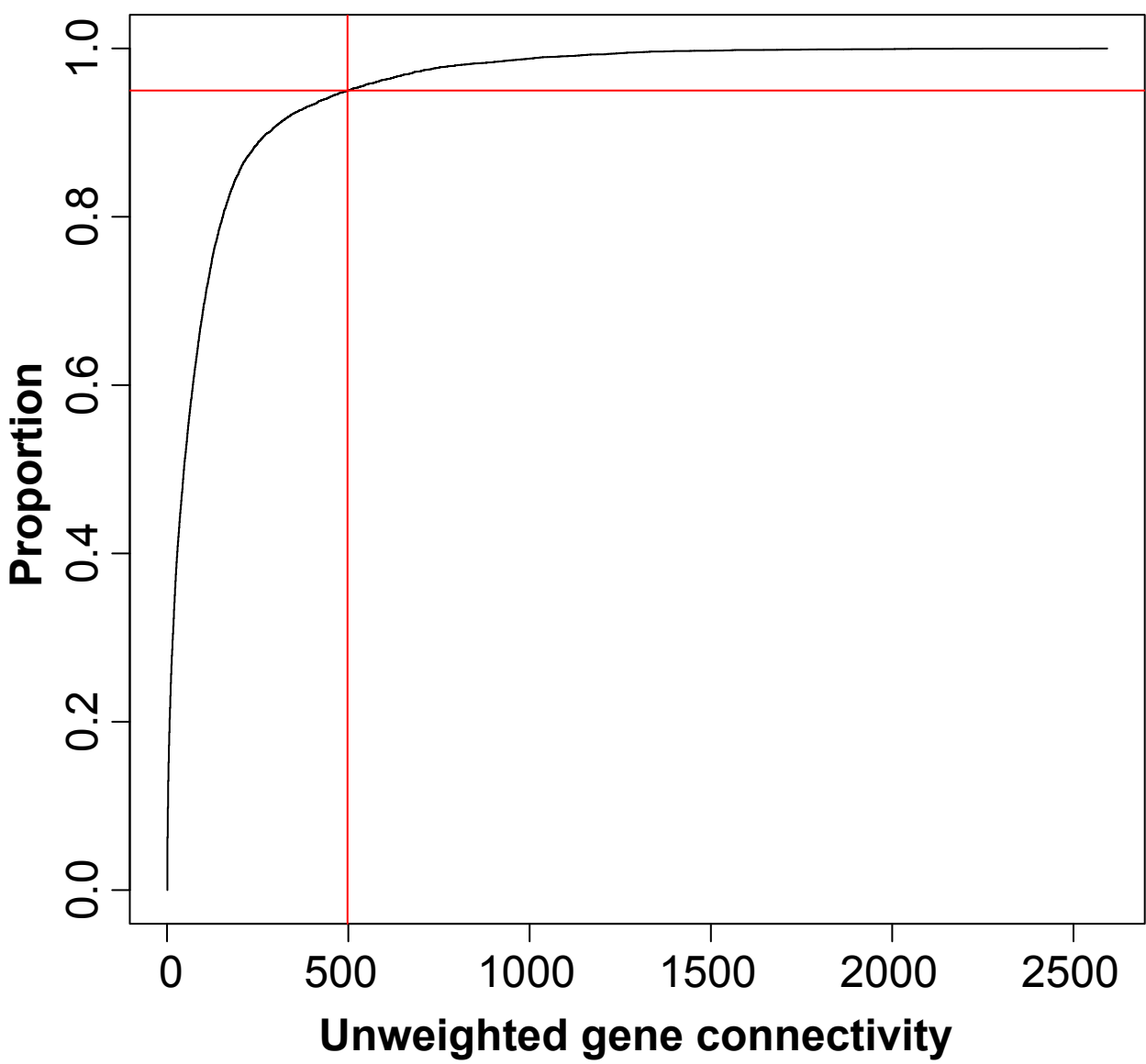

Supplement: S3 Fig — Empirical distribution of the probability (P(k)) of finding nodes containing k edges (nodes with connectivity of TOM > 0.1) indicates a hierarchical and modular structure. (PDF) [file pone.0118731.s003.pdf]

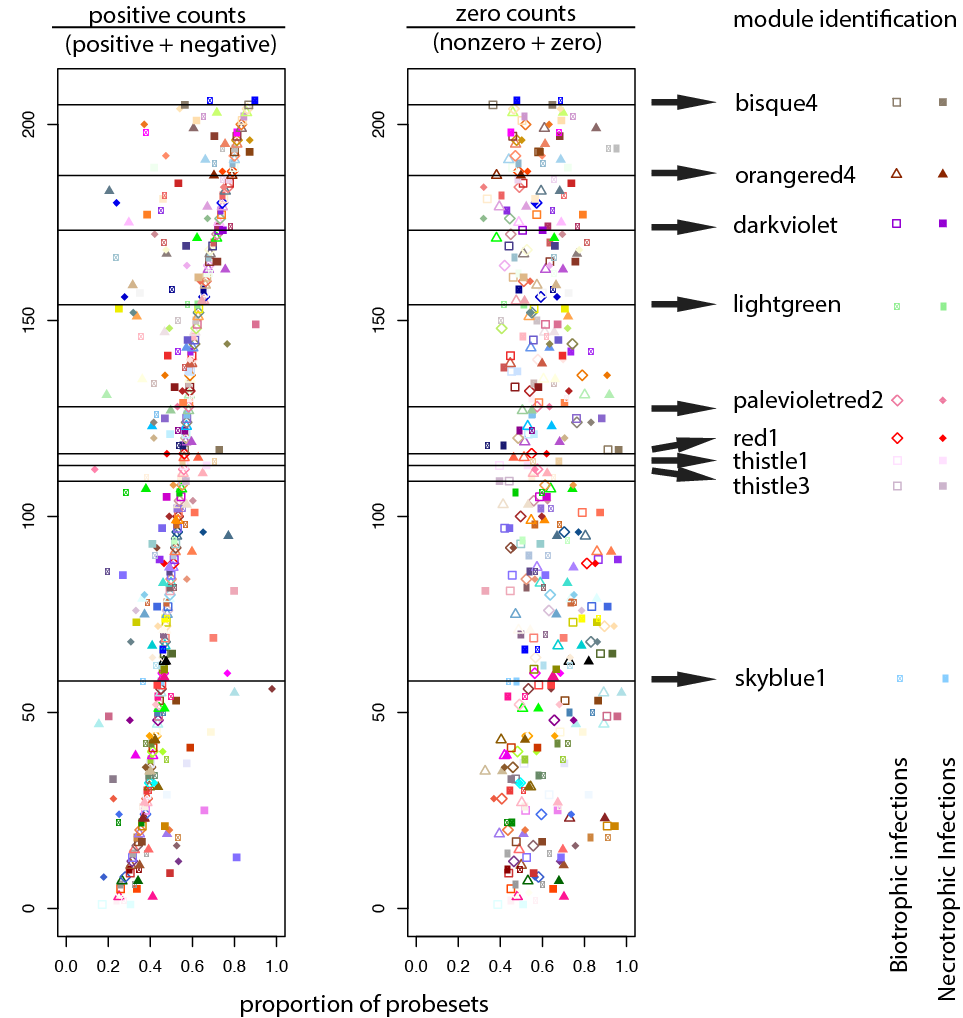

Supplement: S4 Fig — Black lines highlight the ten modules enriched in genes associated with hormone activity. (TIF) [file pone.0118731.s004.tif]

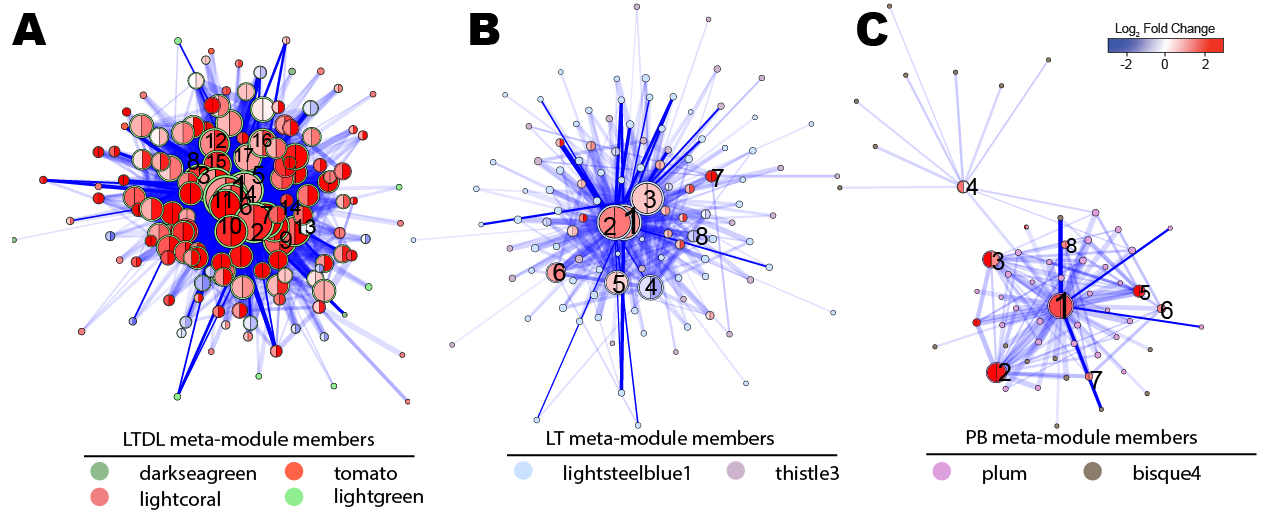

Supplement: S5 Fig — Only nodes with at least one connection with TOM > 0.1 are visualized. Node size is proportional to degree of unweighted connectivity; the colors of the edge of the nodes correspond to module membership; edge width and opacity are proportional to TOM and adjacency values between the two connected nodes, respectively. The central color of each node is based on the mean expression fold-change (up-regulation in red, down-regulation in green) in response to biotrophs (left) and necrotrophs (right). The numbers of nodes correspond to the intra meta-module connectivity ranking (S7 Table). (TIF) [file pone.0118731.s005.tif]
